# Timing of chemotherapy-induced neutropenia: the prognostic factor in advanced pancreatic cancer patients treated with gemcitabine / gemcitabine-based chemotherapy

**DOI:** 10.18632/oncotarget.16980

**Published:** 2017-04-09

**Authors:** Yang Chen, Yan Shi, Huan Yan, Yan Rong Wang, Guang Hai Dai

**Affiliations:** ^1^ Medical Oncology Department 2, Chinese PLA General Hospital and Chinese PLA Medical School, Beijing 100853, China

**Keywords:** advanced pancreatic cancer, timing of chemotherapy-induced neutropenia (CIN), prognosis, chemotherapy

## Abstract

Chemotherapy-induced neutropenia (CIN) was reported to be a predictor of better survival in several cancers. The objective of our study is to evaluate the relationship between the timing (onset) of CIN and prognosis. Between June 2008 and June 2015, 134 patients with confirmed advanced pancreatic cancer received at least one cycle of gemcitabine / gemcitabine-based chemotherapy as first-line chemotherapy were eligible for assessment. Timing of CIN was categorized into early onset and non-early onset CIN group. The end of cycle 2 was the cutoff to differentiate early onset or non-early onset. The correlation between timing of CIN with survival was analyzed by Kaplan-Meier method and Cox proportional hazards model. Median overall survival (OS) was 8.05 months (95% CI: 5.97-10.13) for patients with early onset CIN compared with 5.82 months (95% CI: 5.00-6.63) for patients without early-onset neutropenia (*P* = 0.022). Multivariate analysis proved that timing of CIN was an independent prognostic factor, hazard ratios of death was 0.696 (95% CI: 0.466-0.938) for patients with early onset CIN. In conclusion, timing of CIN is an independent predictor of prognosis in patients with advanced pancreatic cancer undergoing gemcitabine / gemcitabine based chemotherapy. Early-onset CIN predicts better survival.

## INTRODUCTION

Pancreatic cancer is responsible for 331,000 deaths per year, and the seventh most common cause of death from cancer in both genders worldwide [1]. Pancreatic cancer is typically diagnosed late, when curative resection is impossible and prognosis is poor, with only 1-2% of patients surviving at 5 years. Several factors have been proved that could predict survival, such as tumor size, histologic grade, vascular invasion, lymph node metastases and perineural invasion [2]. However, these factors are only available for assessment after surgery. Other novel molecular biomarkers are associated with high costs, time-consuming laboratory experiments. Therefore, there is an urgent need for easily measurable chemotherapy prognostic markers that can be used for stratifying patients’ treatment.

Chemotherapy-induced neutropenia (CIN) is a common adverse effect which often results in dose reduction. Several studies have reported the association of CIN with a better clinical outcome in breast cancer, gastric cancer, cervical cancer, unresectable pancreatic cancer and ovarian cancer patients [3-7]. Massimo Di Maio M *et al* [8] indicated that the occurrence of CIN is associated with better survival in non small cell lung cancer (NSCLC) patients in a pooled analysis. Although a few studies indicated that CIN was correlated to a better survival, the association between severity of CIN (grade of CIN) and prognosis was still controversial. Banerji U *et al* [9] proved that patients experienced grade 3-4 neutropenia had a longer time to progress (TTP) and overall survival (OS) than patients experienced grade 1-2 neutropenia in small cell lung cancer (SCLC). Whereas, in Yamanaka T *et al* study [10], the gastric cancer patients with severe (grade 3-4) neutropenia did not indicate a better survival.

Recently, Jang SH *et al* [11] raised a new viewpoint on the correlation between CIN with survival in their study. They found that timing of CIN might be a predictive factor for favorable prognosis in patients with metastatic NSCLC. The predictive or prognostic role of timing of CIN has not been established in advanced pancreatic cancer. The objective of this study is to investigate the possible correlation between timing of CIN with prognosis in advanced pancreatic cancer patients.

## RESULTS

### Demographics

Total 134 patients with histologically confirmed advanced pancreatic cancer who received at least one cycle of chemotherapy were eligible for assessment. The median age at diagnosis was 57 years. A total of 30 (22.4%) patients were locally advanced and 104 (77.6%) patients had distant metastasis. Among them, 86 (64.2%) patients experienced early onset CIN and 48 (35.8%) patients experienced non-early onset CIN. Table 1 shows clinical characteristics by timing of CIN during the first-line chemotherapy of all patients.

**Table 1:** Advanced pancreatic cancer patients’ characteristics by timing of CIN

Characteristics	Number of patients (%) (*n* = 134)	Early onset *N* = 86	Non-early onset *N* = 48	*P* value
**Age**				
≤57	73(54.5)	47	26	0.957
>57	61(45.5)	39	22	
**Gender**				
Male	87(65)	51	36	0.068
Female	47(35)	35	12	
**KPS**				
90	110(82)	67	43	0.091
70-80	24(18)	19	5	
**Pathological differention**				
Well-moderate	92(69)	53	39	0.070
poor	42(31)	33	9	
**Disease extension**				
Locally advanced	30(22.4)	18	12	0.588
Distant metastasis	104(77.6)	68	36	
**Location**				
Head	44(32.8)	29	15	0.770
Body-tail	90(70.2)	57	33	
**First-line chemotherapeutic regimens**				
Gemcitabine monotherapy	40(29.9)	22	18	0.084
Gemcitabine and S-1/capecitabine	19(14.2)	9	10	
Gemcitabine and platinum drugs	6(4.5)	4	2	
Gemcitabine and nab-paclitaxel	69(51.5)	51	18	
**Second-line chemotherapy**				
Yes	36(27)	24	12	0.716
No	98(73)	62	36	

Tests used: Wilcoxon test; Pearson test.

### Survival analysis

We subsequently evaluated prognostic significance of timing of CIN using Kaplan-Meier survival analysis, univariate and multivariate Cox regression analysis. The median OS for all patients was 7.06 months, 116 (86.5%) patients died by end of the follow-up (July 31^st^, 2016). Patients with early-onset CIN had a longer survival than those non-early onset CIN (median OS: 8.05m vs. 5.82m, *P* = 0.022) (Figure 2)

**Figure 1:**
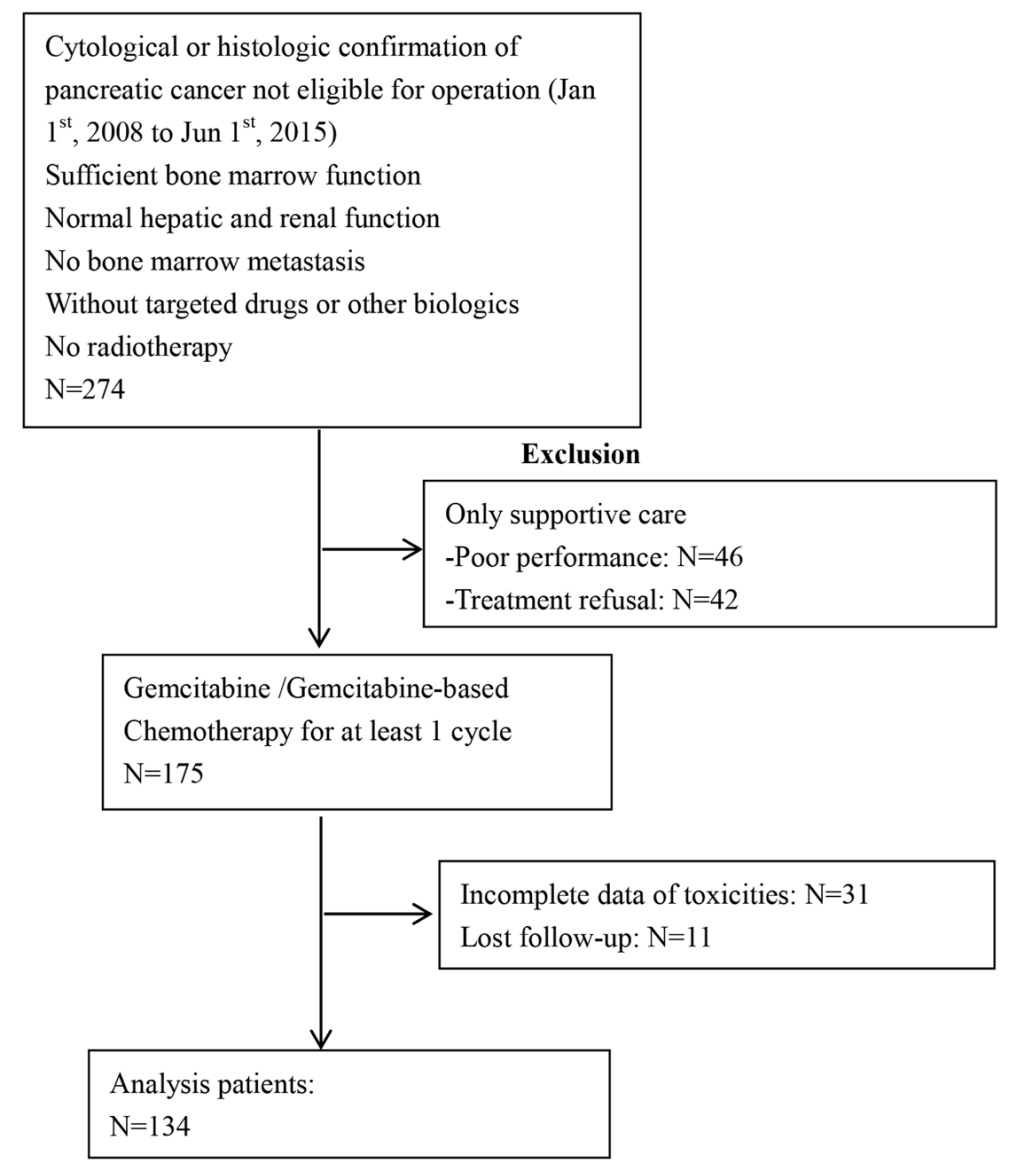
Study flow chart

**Figure 2:**
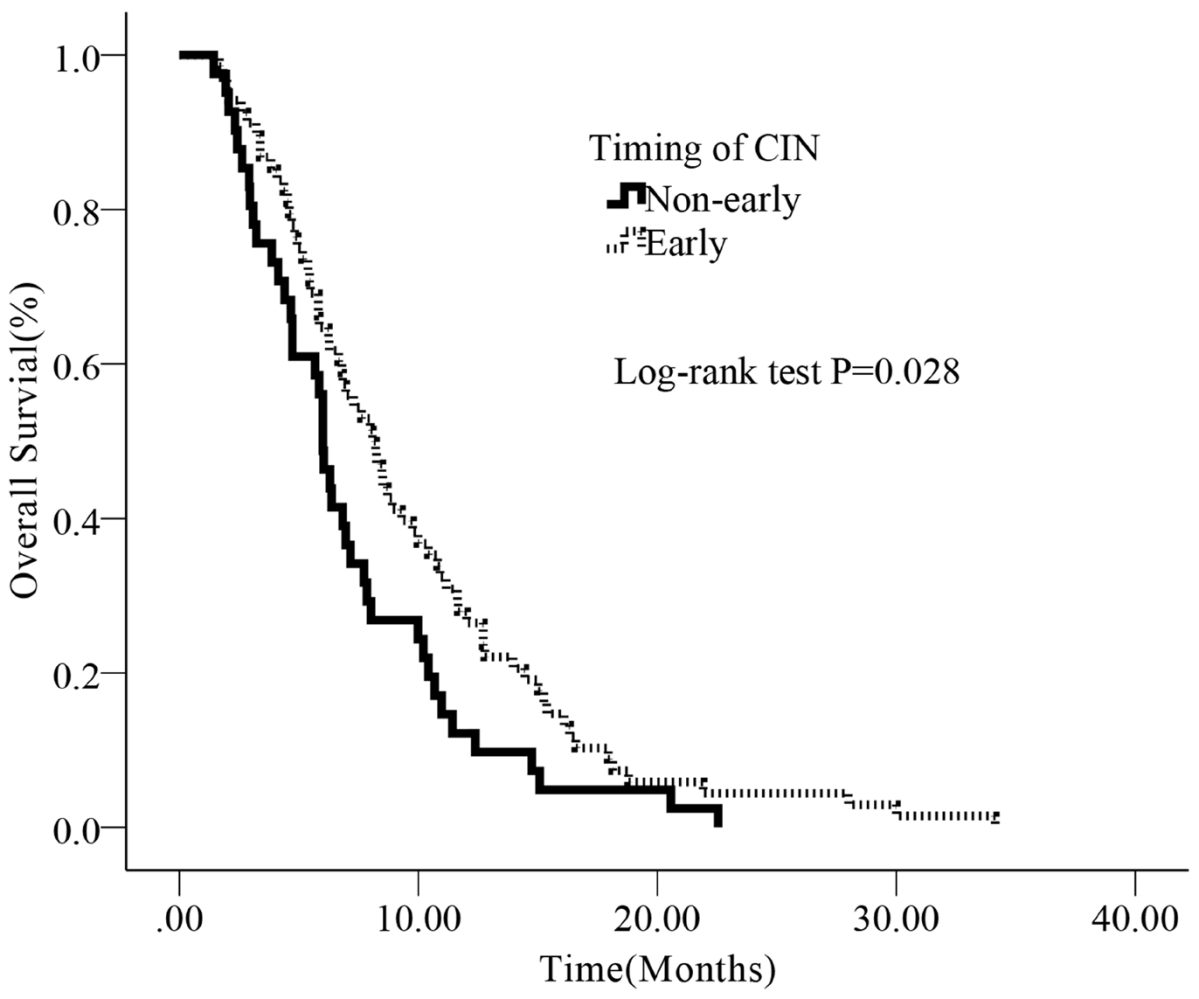
Kaplan-Meier survival curves according to timing of CIN for advanced pancreatic cancer patients The median OS in the early onset group and non-early onset group were 8.05 months (95%CI: 5.97-10.13) and 5.82 months (95% CI: 5.00-6.63), respectively.

Univariate analysis identified female (female vs. male, *P* = 0.018), a better tumor pathological differention (poor vs. well-moderate, *P* = 0.043), patients received gemcitabine and nab-paclitaxel chemotherapy (gemcitabine and nab-paclitaxel vs. gemcitabine monotherapy, *P* = 0.016), patients with early onset CIN (early onset vs. non-early, *P* = 0.023) and patients received second-line chemotherapy (yes vs. no, *P* = 0.019) as better prognostic factors for OS in this study cohort. Age, KPS, location, stage were not significantly associated with clinical outcome (Table 2).

**Table 2:** Univariate analysis for the association between clinical characteristics with survival in advanced pancreatic cancer patients

	HR	95%CI	*P* value
**Gender**			
Male	1		
Female	0.617	0.420-0.907	0.018
**Age**			
≤57	1		
>57	1.044	0.615-1.774	0.873
**KPS**			
90	1		
70-80	2.376	0.868-6.499	0.092
**Pathological differention**			
Well-moderate	1		
Poor	1.132	0.954-1.343	0.043
**Location**			
Head	1		
Body/tail	1.013	0.694-1.480	0.946
**Disease extension**			
Distant metastasis	1		
Locally advanced	0.693	0.447-1.074	0.101
**First-line chemotherapeutic regimens**			
Gemcitabine monotherapy	1		
Gemcitabine and S-1/capecitabine	0.783	0.450-1.363	0.387
Gemcitabine and platinum drugs	0.522	0.219-1.244	0.142
Gemcitabine and nab-paclitaxel	0.601	0.397-0.910	0.016
**Second-line chemotherapy**			
No	1		
Yes	0.666	0.474-0.936	0.019
**Timing of CIN**			
Non-early	1		
Early	0.644	0.441-0.941	0.023

Abbreviation: CIN, chemotherapy-induced neutropenia;

A two-sided significance level of 0.05 was used to evaluate statistical significance.

To determine the independent prognostic value of timing of CIN for OS, a multivariate analysis using a Cox proportional hazard model was performed. In the multivariate analysis that included gender, age, KPS, pathology differention, disease extension, first-line chemotherapy regimens, second-line chemotherapy and timing of CIN, we identified timing of CIN (early onset vs. non-early, HR: 0.696 [0.466-0.983] , *P* = 0.027), pathological differention (poor vs. well-moderate, HR: 1.159 [1.078-1.417], *P* = 0.037) and second-line chemotherapy (yes vs. no, HR: 0.681 [0.476-0.972] , *P* = 0.035) were independent prognostic factors for OS (Table 3).

**Table 3:** Multivariate analysis for the association between clinical characteristics with survival in advanced pancreatic cancer patients

	HR	95%CI	P value
**Gender**			
Male	1		
Female	0.680	0.443-1.043	0.177
**Age**			
≤57	1		
>57	1.099	0.982-1.016	0.914
**KPS**			
90	1		
70-80	1.174	1.037-1.419	0.049
**Pathological differention**			
Well-moderate	1		
Poor	1.159	1.078-1.417	0.037
**Disease extension**			
Distant metastasis	1		
Locally advanced	0.761	0.467-1.012	0.087
**First-line chemotherapeutic regimens**			
Gemcitabine monotherapy	1		
Gemcitabine and S-1/capecitabine	0.966	0.548-1.812	0.990
Gemcitabine and platinum drugs	0.929	0.363-2.374	0.878
Gemcitabine and nab-paclitaxel	0.846	0.520-1.376	0.501
**Second-line chemotherapy**			
No	1		
Yes	0.681	0.476-0.972	0.035
**Timing of CIN**			
Non-early	1		
Early	0.696	0.466-0.938	0.027

Hazard ratios of survival and 95% CI were estimated with Cox’s proportional hazards model.

Adjusted for: Gender, Age, KPS, Pathology differention, Disease extension, First-line chemotherapy regimens, Second-line chemotherapy, Timing of CIN

## DISCUSSION

This is the first study, to our knowledge, to prove timing of CIN is a predictor of better prognosis in advanced pancreatic cancer patients undergoing gemcitabine / gemcitabine-based chemotherapy. In our study, median OS was 8.05 months (95% CI: 5.97-10.13) for patients with early onset CIN compared with 5.82 months (95% CI: 5.00-6.63) for patients without early-onset CIN (*P* = 0.028). Multivariate analysis proved timing of CIN was an independent prognostic factor.

Our study showed that early-onset CIN group had a significantly better OS than non-early CIN patients, which is consistent with the conclusion by Jang SH et al, who divided 123 NSCLC patients into early-onset group (within 2 cycles), late-onset group (3-6 cycles) and absence. They found that early-onset neutropenia group showed significantly better PFS and OS than the late-onset group but there was no difference between patients with late-onset and absence of neutropenia group. Intriguingly, it was a new finding that the timing (onset) is a prognostic factor.

As we all known, the majority of patients could experience neutropenia during chemotherapy. Some of them might have better survival than those without CIN. Although CIN certainly is not a direct reason for favorable outcome, some studies as well as our findings suggested that CIN might be an indicator for a) the biological activity of cytotoxic drugs, b) patient’s genetic predisposition of cytotoxic drugs and c) inflammation level of the patient, which were common factors related to prognosis.

First, the therapeutic effects of patients depend on sufficient biological activity of cytotoxic drugs reaching cancer cell. Although chemotherapy drugs dosing is based on patients’ estimated body surface area (BSA)[12, 13], this method has been questioned because of uncertain relationship between BSA and the pharmacokinetics of most cytotoxic agents[14]. Plasma concentration of drugs is affected by patients’ different metabolisms, distribution, and catabolism (hepatic and renal blood flow, activity of enzymatic systems). Two decades ago, Gurney H *et al* [12] displayed the limitations of BSA dosing which does not account for the whole complex process of cytotoxic drug metabolism. This can lead to an under-dosing of nearly 30% patients who may have poor oncologic outcome. Moreover, assessment of drug plasma concentration in every patient is too expensive and not practical. Therefore, it might be more convenient to use CIN instead of drug plasma concentration as a predictive factor of efficacy after prospective study validates the results of our study.

Second, the sensitivity of tumor cells to therapeutic drugs is a reflection of the patients’ genetic predisposition[15], and theoretically sharing similar features of pharmacokinetics and pharmacodynamics of the regimen in all cells in this patient[14]. Our results showed that early onset CIN indicated a better treatment response, which suggested that patients with early onset neutropenia might be the sensitive population to gemcitabine / gemcitabine-based chemotherapy. On the other hand, multidrug resistance is known as the cross-resistance of cancer cells to various cytotoxic drugs which are structurally or functionally unrelated [16]. Either intrinsic resistant or acquired resistant could produce chemotherapeutic failure and malignant tumor progression in cancer [17]. The non-early onset CIN group patients might be resistant to gemcitabine-based chemotherapy intrinsically, and even insensitive to other cytotoxic regimens result in unfavorable outcome. Neutrophil count may be influenced by a host of clinical factors besides performance status, such as age, prior treatments, coexisting infection, and impaired renal or hepatic function [18]. Our multivariate analysis showed strong protective effects of early-onset CIN.

It is not fully understood why such a relationship exists. Although a recent analysis failed to correlate CIN with DNA repair genes, the prognostic significance of CIN may derive from cooperative effects of the repair genes[19]. Thus, patients could respond differently to anti-cancer drugs. The application of neutropenia as a pharmacokinetic marker could be used to individualize the biological effect of patients. Lack of neutropenia may suggest weak biological effect of chemotherapy, which is possible due to a low dose for an individual patient or patients’ intrinsic resistant.

Furthermore, inflammation plays an important role in development and progression of tumor [20]. Changes in hematology ingredients like white blood cells, specifically the neutrophils have been made because of systemic inflammatory response[21]. Neutrophils could promote the acceleration of angiogenesis and suppress the anti-tumor immune response, thereby speed up tumor proliferation [22-24]. In other words, elevated neutrophil counts could promote tumor progression by providing an advantageous environment for proliferation. Jensen HK *et al* reported that the elevated neutrophils were significantly associated with larger tumor size and worse survival in patients with localized renal cell carcinoma [25]. Another study including 1533 patients with nasopharyngeal cancer (NPC) found that elevated neutropenia predicted poor prognosis [26].Therefore, there is another possible hypothesis for explaining our results that early-onset CIN, decreased neutrophil counts earlier induced by chemotherapy, might change tumor environment by disrupting angiogenesis and releasing immune suppression, and result in favorable clinical outcome.

Based on the three possible mechanisms mentioned above, theoretically occurrence of CIN, especially for early-onset CIN, might be a predictive factor for favorable prognosis [14, 27]. The significant marker achieved by univariate analysis need to be further validated in the multivariate analysis. Therefore, we think gender is not an independent prognostic factor. In our study, patients with second-line chemotherapy is an independent prognostic factor, there might be the possibility that duration of chemotherapy itself might affect the treatment results. More frequently, most patients could not have the opportunity to receive the second-line chemotherapy due to disease progression, which might be due to the more invasive biological behavior of the tumor itself. Therefore, we think it is reasonable that second-line chemotherapy might be an independent prognostic factor.

This study has a few limitations: the retrospective nature, the limited sample size, restriction to Chinese population and confined the chemotherapy to gemcitabine / gemcitabine based chemotherapy. Despite these above, our results have confirmed the use of a potential clinical biomarker (timing of CIN) in predicting clinically outcomes. It also has some clinical applications including predicting patients’ chemotherapy response, and prognosis, adjusting drug dosage or taking anti-inflammatory mediators.

In conclusion, our study suggested that early-onset CIN was a predictive factor for favorable prognosis in patients with advanced pancreatic cancer. We provide a simple, convenient and potential option, using timing of CIN as an indicator, to predict patient’s response to chemotherapy early and individualize optimum dosing of drugs. More well-designed and large-scale investigations are warranted to better understand the value of timing of CIN in prognosis of cancer patients.

## MATERIALS AND METHODS

### Patients

The study was comprised of advanced pancreatic cancer patients admitted to Chinese People’s Liberation Army (PLA) General Hospital from January 1^st^_,_ 2008 to June 1^st^_,_ 2015. Our study was approved by the ethics committee of PLA General Hospital. Before the initial time of chemotherapy, written informed and consent were submitted from the patients or their legal guardian. All treatments were performed in accordance with relevant guidelines and regulations. Blood samples were collected under institutional review board-approved protocols. Clinical data retrieved electronically from the medical records of PLA General Hospital Registry were reviewed retrospectively.

The inclusion criteria were : 1) patients with cytological or histologic confirmation of pancreatic cancer without any chance of radical operation (including locally advanced and distant metastasis); 2) patients received at least one cycle of gemcitabine / gemcitabine-based chemotherapy as first-line treatment; 3) patients’ absolute neutropenia count (ANC) >2.0×10^9^/L before treatment; 4) sufficient bone marrow function; 5) normal hepatic and renal function; 6) KPS≥70; 7) without targeted drugs or other biologics; 8) no bone marrow metastasis; 9) no history of prior chemotherapy for advanced disease or adjuvant therapy within one year; 10) no radiotherapy. Exclusion criteria: 1) no integrated data for toxic effects; 2) no follow-up result. Follow-up evaluations were performed every 3 months. Dates of death were obtained from the China disease prevention and control information system or telephone calls to their families. Medical records were reviewed, and the cause of death was assigned by study physicians. Loss to follow-up refers to patients who became unreachable. We followed up until June 31st, 2016 to obtain clinical and outcome information. Specific details of enrollment and exclusion were also showed in the following flow chart (Figure 1).

### Dose intensity of chemotherapy

Patients in this study all received gemcitabine / gemcitabine-based chemotherapy. Patients had undergone at least one cycle of gemcitabine / gemcitabine (1000 mg/m^2^, d1, 8, every 3-week)-based chemotherapy as first-line chemotherapy,

### Assessment of neutropenia

Complete blood cell (CBC) count was checked prior to infusion of agents (Days 1 and 8) and every 7 days. ANC was computed by multiplying the white blood cell count by the total percentage of neutrophils. CIN grading was in accordance with National Cancer Institute (NCI) Common Terminology Criteria for Adverse Events (CTCAE, version 4.0).CIN was also categorized according to time of onset. Early-onset neutropenia was defined as the lowest ANC<2.0×10^9^/L before the end of cycle 2, non-early onset CIN defined as the patients had no experience of ANC<2.0×10^9^/L or onset on the end of cycle 2 or after.

### Assessment of survival

OS was defined from date of treatment to death. The primary study endpoint was OS. Censoring occurred if patients were still alive at last follow-up or dead from other diseases.

### Statistical analysis

Data was presented as median (interquartile) for continuous variables, and as frequency or percentage for categorical variables. The Mann-Whitney and chi-square tests were used to determine any statistical difference between the proportions of the two groups. Survival curves of each category were estimated by the Kaplan-Meier method and compared by the log-rank test. Hazard ratios of survival and 95% CI were estimated with Cox’s proportional hazards model. In COX model we adjusted for: KPS; Pathological differention; disease extension; second-line chemotherapy; timing of CIN.

All of the analyses were performed with the statistical software packages R (http://www.R-project.org, The R Foundation) and EmpowerStats (http://www.empowerstats.com/en/, X&Y Solutions, Inc., Boston, MA). A two-sided significance level of 0.05 was used to evaluate statistical significance.

## SUPPLEMENTARY MATERIALS FIGURE


