# Supplementary material for: Timing of chemotherapy-induced neutropenia: the prognostic factor in advanced pancreatic cancer patients treated with gemcitabine / gemcitabine-based chemotherapy
Source: Oncotarget. 2017 Apr 9;8(39):66593–600. doi: 10.18632/oncotarget.16980 (PMC5630440; doi:10.18632/oncotarget.16980)
Supplement: Supplementary file 1 [file oncotarget-08-61404-s001.pdf]

Timing of chemotherapy-induced neutropenia: the prognostic factor in advanced pancreatic cancer patients treated with gemcitabine / gemcitabine-based chemotherapy

Supplementary Material

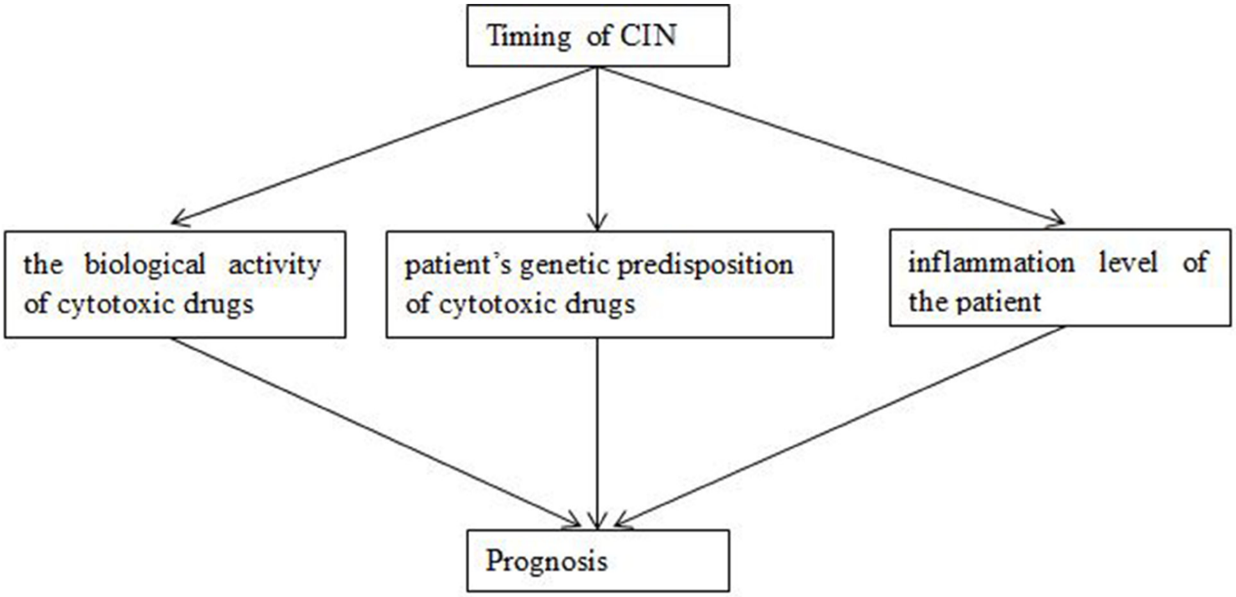

Supplementary Figure 1: Potential mechanisms for timing of CIN as a prognosis factor
